# Epidemiology of extended-spectrum β-lactamases in *Enterobacterales* in Taiwan for over two decades

**DOI:** 10.3389/fmicb.2022.1060050

**Published:** 2023-01-25

**Authors:** Chien-Ming Chao, Chih-Cheng Lai, Wen-Liang Yu

**Affiliations:** ^1^Department of Intensive Care Medicine, Chi Mei Medical Center, Liouying, Taiwan; ^2^Department of Dental Laboratory Technology, Min-Hwei College of Health Care Management, Tainan, Taiwan; ^3^Division of Hospital Medicine, Department of Internal Medicine, Chi Mei Medical Center, Tainan, Taiwan; ^4^Department of Intensive Care Medicine, Chi Mei Medical Center, Tainan, Taiwan; ^5^Department of Medicine, School of Medicine, College of Medicine, Taipei Medical University, Taipei, Taiwan

**Keywords:** *Enterobacterales*, ESBL, *Escherichia coli*, *Klebsiella pneumoniae*, *Proteus mirabilis*

## Abstract

The emergence of antimicrobial resistance among microorganisms is a serious public health concern, and extended-spectrum β-lactamases (ESBL)-producing *Enterobacterales* is one of the major concerns among antibiotic-resistant bacteria. Although the prevalence of ESBL in *Enterobacterales* has been increasing with time, the prevalence of ESBL could differ according to the species, hospital allocation, sources of infections, nosocomial or community acquisitions, and geographic regions. Therefore, we conducted a comprehensive review of the epidemiology of ESBL-producing *Enterobacterales* in Taiwan. Overall, the rates of ESBL producers are higher in northern regions than in other parts of Taiwan. In addition, the genotypes of ESBL vary according to different *Enterobacterales*. SHV-type ESBLs (SHV-5 and SHV-12) were the major types of *Enterobacter cloacae* complex, but *Serratia marcescens, Proteus mirabilis, Escherichia coli,* and *Klebsiella pneumoniae* were more likely to possess CTX-M-type ESBLs (CTX-M-3 and CTX-M-14). Moreover, a clonal sequence type of O25b-ST131 has been emerging among urinary or bloodstream *E. coli* isolates in the community in Taiwan, and this clone was potentially associated with virulence, ESBL (CTX-M-15) production, ciprofloxacin resistance, and mortality. Finally, the evolution of the genetic traits of the ESBL-producing *Enterobacterales* isolates helps us confirm the interhospital and intrahospital clonal dissemination in several regions of Taiwan. In conclusion, continuous surveillance in the investigation of ESBL production among *Enterobacterales* is needed to establish its long-term epidemiology.

## 1. Introduction

The rapid development of antimicrobial resistance among microorganisms is a serious public health concern, and there were an estimated 4.95 million deaths associated with antimicrobial resistance. Based on the estimation of the global burden of antibiotic resistance by Antimicrobial Resistance Collaborators, *Escherichia coli* and *Klebsiella pneumoniae* were the first and third leading pathogens associated with resistance. In addition, third-generation cephalosporin-resistant *E. coli* and *K. pneumoniae* would cause 50,000–100,000 deaths. Many antibiotic-resistance mechanisms have been identified among *Enterobacterales* and extended-spectrum β-lactamases (ESBLs), which remained one of the most common mechanisms (Lai et al., 2014; Colmenarejo et al., 2020; Vink et al., 2020; Estaleva et al., 2021; Karlowsky et al., 2022; Sader et al., 2022; Selmi et al., 2022). ESBLs that mediate resistance to newer β-lactam antibiotics, including extended-spectrum cephalosporins and monobactams, are plasmid-mediated class A enzymes commonly found in the family *Enterobacterales* and frequently detected among *E. coli* and *K. pneumoniae*. For adult patients with community-onset bacteremia, ESBL-producing *E. coli*, *Klebsiella* species, and *Proteus mirabilis* pathogens were linked to inappropriate empirical antibiotic therapy and the 4-week mortality (Lee et al., 2017). Therefore, it should be concerned that the ESBL-producing *Enterobacterales* are associated with a high mortality rate and increased medical care costs. Based on the finding of the Regional Resistance Surveillance program that monitored susceptibility rates and developing resistance by geographic region, including 12 Asia-Pacific countries (Mendes et al., 2013), the resistance rates among nations within the area can be diverse, being generally lower in Japan and Australia/New Zealand and higher in eastern Asia countries. In detail, ESBL phenotype rates in *E. coli* and *Klebsiella* spp. were 48 and 47%, respectively, ranging from 11%/10% in New Zealand to 91%/75% in Taiwan (Mendes et al., 2013). Another study focusing on *Enterobacterales* isolates that caused intra-abdominal infections in hospitalized patients in the Asia-Pacific region revealed a higher resistance burden in Vietnam and the Philippines than in other Asia-Pacific countries (Jean and Hsueh, 2017). However, the high prevalence of ESBL producers in Taiwan might be due to selection bias. Therefore, this study aimed to conduct a comprehensive review of the epidemiology of ESBL-producing *Enterobacterales* in Taiwan.

## 2. The general prevalence of ESBL in *Enterobacterales*

The prevalence of ESBL in *Enterobacterales* could be variable according to the species, hospital allocation, sites of infection, as well as nosocomial or community acquisition, however, the prevalence of ESBL was increasing over time in Taiwan (Yu et al., 2006c; Table 1). In intensive care units (ICUs), a surveillance investigation of 574 *Enterobacterales* isolates in 10 major teaching hospitals in Taiwan in 2005 showed the prevalence of ESBL production was 26, 16, 14, and 13% in *K. pneumoniae*, *Serratia marcescens*, *E. coli and Proteus mirabilis*, respectively (Jean et al., 2009). Another surveillance of 336 *Enterobacterales* isolates from patients with intra-abdominal infections in a north medical center between 2000 and 2006 showed the overall prevalence of ESBL production was 26, 23, and 19% among *E. coli*, *Klebsiella* spp., and *Enterobacter* spp. (Chen et al., 2009). During the seven-year study period, the highest rate of ESBL production was found in 2005 for *E. coli* (38%) and in 2003 for *Klebsiella* spp. (38%) and *Enterobacter* spp. (40%). The incidence of ESBL-producing isolates declined in 2005 and 2006 (Chen et al., 2009). Shu et al. reported that the prevalence of ESBL increased from 4.8 to 10.0% during seven-year surveillance in a large teaching hospital in north Taiwan (Shu et al., 2010). Another surveillance of pathogens isolated from patients with complicated intra-abdominal infections at five medical centers from 2006 to 2010 found that the rate of ESBL-producing species was three-fold higher among patients with nosocomial IAIs than among patients with community-acquired IAIs (Lee et al., 2012). Moreover, the rate of ESBL producers was highest in northern Taiwan and lowest in central Taiwan (Lee et al., 2012). One of the reasons for the differences in prevalence of ESBL producers in different regions in Taiwan could be associated with the prescription patterns by physicians. For example, the highest rate ceftazidime resistance among *Pseudomonas aeruginosa* isolates and the highest rates of ESBL producers in northern Taiwan could be attributed to the highest ceftazidime utilization in this region. Likewise, the highest rates of carbapenem resistance among *P. aeruginosa* isolates and the lowest rates of ESBL producers in central Taiwan implying the highest carbapenem utilization in the area (Lee et al., 2012). For bacteremia, a retrospective study in a medical center in southern Taiwan between 2008 and 2013 including 1,141 adult patients with community-onset bacteremia due to *E. coli*, *K. pneumoniae*, and *P. mirabilis* found that only 65 (5.7%) isolates were ESBL producers and were associated with poor prognosis (Lee et al., 2018). Regarding community-onset urinary tract infections (UTIs), a medical center in northern Taiwan reported a prospective study of 393 isolates from urine cultures, including 253 *E. coli* and 42 *K. pneumoniae* isolates. Fifty-three (13.5%) isolates were phenotypically positive for ESBL production (Kung et al., 2015). All these findings suggest that regular monitoring and surveillance investigation of ESBL production among *Enterobacterales* is needed to establish its associated epidemiology.

**Table 1:** Prevalence of extended-spectrum β-lactamases (ESBLs) among *Enterobacterales* in Taiwan.

Study	Sites and setting	Study period	No of isolates	Type of infection	Prevalence
Jean et al. (2009)	ICUs in 10 medical centers in northern, central and southern Taiwan	2005	574	Respiratory tract: 45.1%	*K. pneumoniae* (26%)
				Bloodstream infection: 14.6%	*S. marcescens* (16%)
				Other: 7.8%	*E. coli* (14%)
					*P. mirabilis* (13%)
Chen et al. (2009)	1 medical center in northern Taiwan	2002–2006	336	Intra-abdominal infection	*E. coli* (26%)
					*Klebsiella* spp. (23%)
					*Enterobacter* spp. (19%)
Shu et al. (2010)	1 medical center in northern Taiwan	2001–2007	84,458 *E. coli*	NA	*E. coli* (8.3%)
			31,633 *K. pneumoniae*		*K. pneumoniae* (18.1%)
Lee et al. (2012)	5 medical centers in northern, central and southern Taiwan	2006–2010	1956	Complicated intra-abdomianl infecton	*E. coli* (7.6%)
					*K. pneumoniae* (8.2%)
Lee et al. (2018)	A medical center in southern Taiwan	2008–2013	1,141	Community-onset bacteremia	5.70%
Kung et al. (2015)	Emergency department in a medical center in northern Taiwan	2010–2012	393	Community-onset urinary tract infection	13.50%

## 3. Epidemiology of ESBLs-producing *Enterobacterales* in wastewater and river water

In addition to the clinical settings, we cannot neglect the presence of multi-drug resistant (MDR) organisms in wastewater, which could be a major source of antibiotic-resistant bacteria released into the environment (Gomi et al., 2018). The occurrence of ESBL-producing *Enterobacterales* recovered from hospital wastewater is increasing, and these MDR organisms can be eventually discharged into the environment and might contaminate the food chain (Fadare and Okoh, 2021). In addition, Tao et al. also reported that wastes from animal husbandry could be a potential environmental source of antibiotic-resistant pathogens (Tao et al., 2014). Moreover, one study sampling river water from 40 stations in southern Taiwan found that ESBL-producing *E. coli* strains were commonly isolates, accounting for 30% of the 621 *E. coli* strains (Chen et al., 2016). Further analysis showed that the most commonly resistant mechanism revealed CTX-M group 9 and the proportion of ESBL-producing *E. coli* was significantly higher in regions with a large number of chickens being raised (Chen et al., 2016).

## 4. Risk factors associated with ESBL acquisition in Taiwan

### 4.1. Health carriers

Two studies evaluated the risk factors of healthy carriers with ESBL-producing *Enterobacterales* in Taiwan (Huang Y. S. et al., 2020; Cheng et al., 2022). One prospective cohort study including healthy adults who attended a health examination in the local community reported a high carrier rate of third-generation cephalosporin-resistant *E. coli* or *K. pneumoniae* (27.4% ESBL-producing strains), especially in those being an employee of a technology company [adjusted odds ratio (OR), 4.127; 95% confidence interval (CI), 1.824–9.336], and traveling to Southeast Asia in the past year (adjusted OR, 6.545; 95% CI, 1.071–40.001; Huang Y. S. et al., 2020). However, in another cohort of asymptomatic adults in Taiwan, travel to Asian countries and food habits were not associated with fecal carriage of ESBL-producing *E. coli* isolates (Wu et al., 2019). Another prospective study collected stool samples from children aged 0–18 years within 3 days of hospitalization and found several anthropogenic risk factors including drinking water process, pork consumption, pets, and household density might be associated with ESBL-producing *E. coli* (Cheng et al., 2022).

### 4.2. Hospital and patient factors

In addition to healthy carriers, many factors have been found to be associated with the acquisition of ESBL-producing *Enterobacterales* infections in Taiwan. A case–control study in neonatal ICU found that previous usage of 3rd generation cephalosporin (OR, 4.72; 95% CI, 2.03–10.97) and underlying renal disease (OR, 4.07; 95% CI, 1.10–15.08) were identified as independent risk factors for ESBL acquisition (Tsai et al., 2016). Among children, Kuo et al. reported that recent antibiotic exposure (within 30 days before the episode) was the most important predisposing factor associated with infection of ESBL-producing *K. pneumoniae* (Kuo et al., 2007). Additionally, other possible risk factors included recent surgery, the application of mechanical ventilation, nasogastric tubes, and central venous catheter insertion (Kuo et al., 2007). For elderly patients, multiple underlying comorbidities (OR, 2.88; *p* < 0.05) or receiving more than two antimicrobial agents (OR, 3.71; p < 0.05) were associated with increased risks for acquiring the ESBL-producing microorganisms (Lin et al., 2013). Moreover, tracheostomy (OR, 5.13; 95% CI, 1.24–21.1) and ceftazidime use (OR, 13.40; 95% CI, 1.21–148.85) were independently associated with ESBL-producing *K. pneumoniae* (Lin et al., 2003). The acquisition of ESBL-producing microorganism infections has been reported to be associated with recent hospitalization, hospital-acquired infection, and urinary catheter placement (Hsieh et al., 2010; Wu et al., 2010, 2014a; Lin et al., 2011; Tsui et al., 2012). Finally, nasogastric tube placement and hospitalization within the previous 3 months were significantly associated with the acquisition of ESBL-producing pathogens in community-onset UTIs (Kung et al., 2015).

### 4.3. Antibiotic consumption

Increased antimicrobial consumption and inappropriate empirical use have been presumed associated factors with ESBL production in *Enterobacterales* in Taiwan. A 13-year study in a university hospital demonstrated a significant correlation between antimicrobial resistance and increased antimicrobial consumption of some antibiotics in Taiwan (Hsueh et al., 2005). For example, a significant positive association was found between cefotaxime-resistant *E. coli* and annual consumption of cefotaxime (*r* = 0.764 and *p* = 0.002). Another study showed that adult patients with ESBL-producing *E. coli* bacteremia had more antimicrobial agents used in the previous 3 months (50.0 vs. 11.1%, *p* = 0.017) than did those with non-ESBL-producing *E. coli* bacteremia (Tsai et al., 2018). The diverse antibiotic usage by physicians’ prescription might also have contributed to different rates of ESBL producers among different regions in Taiwan (Lee et al., 2012). This study also showed that more adult patients with ESBL-producing *E. coli* bacteremia received inappropriate initial antimicrobial treatment after hospitalization than those with non-ESBL-producing *E. coli* bacteremia (96.3 vs. 35.7%, *p* < 0.001; Tsai et al., 2018).

## 5. The genotype of ESBL in *Enterobacterales*

The genotypes of ESBL are also different in various studies in Taiwan (Table 2). In 1999, Siu et al. reported that SHV-5 was the main genotype of ESBL and the presence of 11 in 16 ESBL-producing gram-negative bacteria caused nosocomial bacteremia in a pediatric oncology ward in a north Taiwan hospital (Siu et al., 1999). In 2004, Yu et al. first reported CTX-M-15 from 2 *K. pneumoniae* isolates in Taiwan (Yu et al., 2004a). In 2005, Chia et al. demonstrate that SHV-12 (*n* = 80) was the most prevalent genotype of 199 ESBL-producers, followed in order of frequency by CTX-M-3 (*n* = 65) and CTX-M-14 (*n* = 36; Chia et al., 2005). In addition, 17 (9%) clinical isolates harbored both SHV- and CTX-M-type ESBLs. SHV-type ESBL was the major type of *Enterobacter cloacae complex*, but *E. coli* and *K. pneumoniae* were more likely to possess CTX-M-type ESBLs (Chia et al., 2005). In 2006, Yu et al. reported that the most prevalent types of ESBLs were SHV-5, SHV-12, CTX-M-3, and CTX-M-14 in isolates of *K. pneumoniae* and *E. coli*, however, the prevalence differed according to the institutions (Yu et al., 2006c). Furthermore, SHV-12 and CTX-M-3 have been reported as the most common ESBLs in isolates of *E. cloacae complex* and *S. marcescens*, respectively (Yu et al., 2006c). In 2010, surveillance in a regional hospital in central Taiwan found that CTX-M-14 type (53.6%) was the most prevalent ESBL among 69 *E. coli* isolates, while SHV type (57.6%) was the most dominant among 33 *K. pneumoniae* isolates (Lin C. F. et al., 2010). Moreover, the co-existence of two or more kinds of ESBL in a single isolate was observed in 40.6 and 72.7% of *E. coli* and *K. pneumoniae* isolates, respectively (Lin C. F. et al., 2010). In 2017, Jean et al. using the surveillance investigation of *Enterobacterales* isolates from 2008 to 2014 reported that CTX-M-14, CTX-M-55, CTX-M-15, CTX-M-27, and SHV-12 had been the dominant ESBL-producing *Enterobacterales* in Taiwan (Jean and Hsueh, 2017).

**Table 2:** Genotypic distribution of extended-spectrum β-lactamases (ESBLs) among *Enterobacterales* in Taiwan.

Study	Site and setting	Study period	No of isolates	Distribution of major genotypes
Siu et al. (1999)	A pediatric oncology ward in a north Taiwan hospital	1993–1997	12 *K. pneumoniae* 4 *E. coli*	*K. pneumoniae* SHV-5: 7 (58.3%) SHV-2: 5 (41.7%) *E. coli* SHV-5: 4 (100%)
Chia et al. (2005)	ICUs in a medical center in northern Taiwan	2002–2003	91 *K. pneumoniae* 68 *E. coli* 40 *E. cloacae* complex	*K. pneumoniae* CTX-M-3: 33 (36.2%) SHV-12: 30 (33.0%) SHV-5: 17 (18.7) CTX-M-14: 13 (14.3%) *E. coli* CTX-M-3: 24 (35.3%) CTX-M-14: 23 (33.8%) SHV-12: 15 (22.0%) *E. cloacae complex* SHV-12: 35 (87.5%) CTX-M-3: 8 (20%)
Lin C. F. et al. (2010)	A regional hospital in central Taiwan	2005–2006	69 *E. coli* 33 *K. pneumoniae*	*E. coli* CTX-M-14: 53.6% *K. pneumoniae* SHV type: 57.6%
Jean and Hsueh (2017)	8 centers in Taiwan northern, central and southern Taiwan	2008–2014	290 *E. coli* 127 *K. pneumoniae* 68 *Enterobacter* spp.	*E. coli* CTX-M-14: 73 (25.2%) CTX-M-55: 67 (23.1%) CTX-M-15: 51 (17.6%) CTX-M-27: 36 (12.4%) *K. pneumoniae* CTX-M-14: 38 (29.9%) SHV-12: 34 (26.8%) CTX-M-15: 19 (15%) CTX-M-27: 36 (12.4%) *Enterobacter* spp. SHV-12: 7 (10.3%)
Liu et al. (2021)	A teaching hospital in northern Taiwan	2020	8 *E. coli* (in dogs and cats)	CTX-M-1 group: 6 (75%) CTX-M-9 group: 4 (50%)

Generally, SHV-types (SHV-5 and SHV-12) and CTX-M-types (CTX-M-3 and CTX-M-14) were equally prevalent among *K. pneumoniae* isolates, whereas CTX-M-types (CTX-M-3 and CTX-M-14) were highly prevalent in *E. coli* isolates in Taiwan. Currently, CTX-M-55, −17 and −27 were increasingly emerging in Taiwan. Globally, TEM- and SHV-type ESBLs were the predominant families of ESBLs in the past. For example, TEM-3 was very common in France, but TEM-10 was the most prevalent TEM-type ESBL in the USA (Soilleux et al., 1996; Wiener et al., 1999). SHV-type ESBLs were most often found in clinical isolates of *K. pneumoniae* worldwide. At present, CTX-M-type enzymes are the most commonly found ESBL type with the CTX-M-15 variant dominating worldwide, followed in prevalence by CTX-M-14, and CTX-M-27 emerging in many parts of the world, including Japan, Europe, and the US (Matsumura et al., 2016; Merino et al., 2018; Cameron et al., 2021). TEM-type ESBLs have been infrequent in Taiwan and less detected in ESBL-producing *E. coli* and *Klebsiella pneumoniae* in European isolates as the CTX-M-type enzymes become the most prevalent ESBL worldwide (Castanheira et al., 2021).

In 2021, an animal study involving 50 samples of *E. coli* from dogs and cats detected eight ESBL producers with ESBL genes including the CTX-M-1 and CTX-M-9 groups in Taipei in northern Taiwan (Liu et al., 2021). According to the phylogenic study of CTX-M enzymes (Bonnet, 2004), the CTX-M group 1 from human isolates is mainly composed of CTX-M-1, CTX-M-3, CTX-M-10, CTX-M-12, and CTX-M-15. While the CTX-M-9 group mainly includes nine enzymes (CTX-M-9, CTXM-M-13, CYX-M-14, CTX-M-16, CTX-M-17, CTX-M-19, CTX-M-21, CTX-M-27, and Toho-2). CTX-M-55 is a variant of CTX-M-15 with only one amino acid substitution (Ala-80-Val). Both CTX-M-15 and CTX-M-55 belong to the CTX-M-1 group, however, the prevalence of CTX-M-55 has gradually increased probably due to multiple spreading mechanisms in China (Zeng et al., 2021) and is even higher than CTX-M-15 in *E. coli* with community-onset infections, especially in South China (Zhang et al., 2014). Therefore, it is a warning for the emerging CTX-M-55 as potential wide spreading in Taiwan.

## 6. ESBL prevalence of specific pathogens

The following sections review and discuss specific pathogens commonly isolated in Taiwan, including *E. coli*, *K. pneumoniae*, *E. cloacae* complex, *S. marcescens*, and *P. mirabilis*.

### 6.1. Escherichia coli

In Taiwan, the prevalence of ESBL production has been reported in many studies, however, the prevalence can vary according to different settings, infection sources, and patient groups (Biedenbach et al., 1999).

#### 6.1.1. Prevalence of ESBL-producing *Escherichia coli* in specific allocation

Table 3 summarized the prevalence of ESBL-producing *E. coli* according to specific allocation. In ICU, Hsueh et al. conducted an analysis of clinical specimens from patients in five major teaching hospitals in 2020 and found that ESBL was found in 11.9% of *E. coli* (Hsueh et al., 2001). Further study by Shu et al. showed the prevalence of ESBL increased from 4.8 to 10.0% during seven-year surveillance in a large teaching hospital in north Taiwan (Shu et al., 2010). Further analysis of the distribution of ESBL-producing isolates in different ICUs showed that the most significant increase occurred in medical ICUs, with a peak prevalence rate of 35.9% in 2006 for *E. coli* (Shu et al., 2010). In a respiratory care center, one retrospective analysis in a tertiary care center from January 2001 to December 2002 reported that the ESBL phenotype was found in 31.4% of *E. coli* (Lee C. M. et al., 2009). In the respiratory care ward involving patients who required prolonged or long-term mechanical ventilation, Lin et al. reported that the prevalence of ESBL-producing isolates of *E. coli* was 39.5% (Lin et al., 2013). For nursing home residents, a retrospective study conducted in medical wards of a district hospital in southern Taiwan between July 2009 and June 2011 reported that 52.7–69.5% of *E. coli* isolates had an ESBL (Liu et al., 2016). Due to chronic exposure to multiple antibiotics, *E. coli* isolates from a nursing home, respiratory care center, or respiratory care ward seemed to have higher ESBL rates than those from an acute ICU setting.

**Table 3:** Prevalence of extended-spectrum β-lactamases (ESBLs) among *E. coli* in specific allocation.

Study	Site and setting	Study period	No of isolates	Prevalence of ESBL-producing *E. coli*
Hsueh et al. (2001)	ICUs in five centers in northern and southern Taiwan	2000	177	11.9%
Shu et al. (2010)	ICUs in 1 medical center in northern Taiwan	2001–2007	NA	35.9% (peak in medical ICU in 2016)
Jean et al. (2009)	ICUs in 10 medical centers in northern, central and southern Taiwan	2005	160	14%
Lee C. M. et al. (2009)	Respiratory care center in a medical center in northern Taiwan	2001–2002	51	31.4%
Lin et al. (2013)	Respiratory care wards in northern Taiwan	2006–2007	NA	39.5%
Liu et al. (2016)	Nursing home in southern Taiwan	2009–2011	288	52.7% (urine), 69.6% (sputum), 60.5% (blood)

#### 6.1.2. Infection source

Among the 404 clinical specimens from patients with community-onset *E. coli* bacteremia in a medical center in southern Taiwan, Hsieh et al. identified only 19 (4.7%) isolates were ESBL producers (Hsieh et al., 2010). For premature babies with *E. coli* bacteremia, a small study including 27 cases showed five (18.5%) isolates were ESBL producers (Chen I. L. et al., 2017). Additionally, this study found that the level of serum alanine aminotransferase was significantly lower in the ESBL-producing *E. coli* group than that in the non-ESBL-producing *E. coli* group (Chen I. L. et al., 2017).

Regarding the genotype of *E. coli* causing bacteremia, one study in northern Taiwan including 60 patients with ESBL-producing *E. coli* bacteremia detected 41 (68.3%) isolates with CTX-M β-lactamases. CTX-M-14 accounted for 31 (75.6%) and CTX-M-3 for 9 (22.0%) of the 41 CTX-M isolates, which were associated with chronic renal failure and ICU stay (Wu et al., 2011). In contrast, the study in southern Taiwan found CTX-M group 9 was the most common genotype causing *E. coli* bacteremia, especially in pediatric patients (85.7%), and there were more *E. coli* ST131 in ESBL isolates than in non-ESBL isolates (Tsai et al., 2018). Finally, one retrospective study enrolled 121 adults from southern Taiwan with ESBL-producing *E. coli* bloodstream infections to investigate their sequence type and virulence factors and showed that positivity for the virulence genes iha, hlyD, sat, iutA, fyuA, malX, ompT, and traT was associated with ST131 positivity (all *p* < 0.05; Hung et al., 2019). Moreover, iroN positivity was associated with 30-day mortality among bacteremia patients without UTIs (Hung et al., 2019).

*E. coli* was the most common pathogen causing UTIs. For young patients (0–18 years) with UTI, a 10-year study reported that the ESBL rate had increased from 2% in 2003 to 11% in 2012 (Chen et al., 2014). Another study involving young children aged from 1 day to 36 months reported that the ESBL rate was 3.3% (*n* = 13) among 421 *E. coli* isolates and it accounted for 93.3% of ESBL-producing *Enterobacterales* (Wu et al., 2016). In contrast, another study reported that the prevalence of UTIs due to ESBL-producing *E. coli* in hospitalized children with community-onset UTIs only increased slightly from 0.59% in 2002 to 0.96% in 2006, however, children with ESBL-producing *E. coli* UTIs had a longer hospital stay (*p* = 0.031) than those without (Fan et al., 2014). But a retrospective of 159 infant patients with UTIs found that most of them had no prior history of illness, and no significant risk factors for acquiring ESBL-producing *E. coli*, such as prior antimicrobial use, hospitalization, UTI, and underlying renal diseases (Cheng et al., 2016). For adult patients, a prospective study included 136 patients with gram-negative bacilli causing community-onset UTIs between August 2009 and January 2012 showed that nine (8.0%) of 111 *E. coli* isolates had ESBL production (Wu et al., 2014a).

Intra-abdominal infection was another common type of *E. coli* infection. Surveillance of the pathogens isolated from patients with complicated intra-abdominal infections at five tertiary-care hospitals in Taiwan during the period 2006 to 2010 showed that ESBL production was detected in 7.6% (71/935) of *E. coli* isolates, and the rate of ESBL production in *E. coli* was steady (Lee et al., 2012). Moreover, the rate of ESBL production among *E. coli* isolates collected from patients with nosocomial IAI was higher than those from community-acquired IAI (14.6 vs. 4.7%).

#### 6.1.3. Sequence type 131

ESBL of ST131 has emerged in many parts of the world since the 2000s, including the US, UK, France, Japan, Canada, India, Kuwait, France, Switzerland, Portugal, and Spain (Coque et al., 2008; Komatsu et al., 2018; Day et al., 2019; Broussier et al., 2020; Duffy et al., 2022). There is no exception for Taiwan. The estimated overall ST131 prevalence in this community UTI cohort increased from 11.2% (in 2002–2004) to 17.4% in 2014–2016 (*p* < 0.01). Especially ST131 with ESBL phenotype (cefotaxime-resistant group) increased from 33.3% in 2002–2004 to 72.1% in 2014–2016 (*p* < 0.01; Wang et al., 2021).

#### 6.1.4. Association between ST131 and CTX-M-15

In some countries, including the US and Korea, the multidrug-resistant ST131 clone producing CTX-M-15 has emerged as a major clone in both the community and hospital (Peirano et al., 2010; Park et al., 2012; Cho et al., 2015). However, the study about the association between ST131 and CTX-M-15 in Taiwan is scarce. A study of 122 patients with ESBL-producing *E. coli* bacteremia during a 6-year period from 2005 to 2010 in southern Taiwan reported that the most common ST was ST131 (29.5%, *n* = 36). CTX-M-producing isolates were identified in 72.2% (26/36) ST131 isolates, including CTX-M-14 (*n* = 13), CTX-M-15 (*n* = 9) and CTX-M-3 (*n* = 4; Chung et al., 2012). In addition, CTX-M has been the most common ESBL in *E. coli* from Taiwan and in UTI causing ST131 *E. coli* (Wang et al., 2021). CTX-M-type ESBL gene was detected in 83.5% (66/79) of the ST131 isolates. Among them, ST131 isolates carrying group 1 CTX-M ESBL have emerged in 2014–2016, especially in 2016 (Wang et al., 2021). Since CTX-M-15 belongs to group 1 CTX-M, it is possible that CTX-M-15 might be associated with Taiwanese ST131 *E. coli* clinical isolates, albeit with no further precise identification in the report (Wang et al., 2021).

#### 6.1.5. Adult patients

One study using the database of healthy inhabitants attending health examinations at a medical center in southern Taiwan in 2017 reported that the prevalence rate of asymptomatic ESBL-producing *E. coli* fecal carriage in adults was 1.9% (14/724; Wu et al., 2019). In this study, ST131 was found in 22 (3.0%) adults, and underlying cancer and stroke were associated with ST131 *E. coli* fecal carriage (Wu et al., 2019). Although the ST131 *E. coli* fecal carriage rate was low among asymptomatic adults in this study (Wu et al., 2019), the prevalence of ST131 among ESBL-producing *E. coli* was much higher in the following clinical entities. Wu et al. conducted a retrospective analysis of 371 consecutive community-onset non-ESBL producing *E. coli* bloodstream infections in a 1,200-bed hospital in southern Taiwan in 2010 and found that 60 (16.2%) patients belonged to the ST131 group and clonal group (O25b-ST131) accounted for 5.9% of total isolates (Wu et al., 2014b). In the same institution, Chung et al. showed that ST131 remained the major sequence type, which accounted for 29.5% (36/122) among adult patients with ESBL-producing *E. coli* bacteremia (Chung et al., 2012). In this retrospective study from 2005 to 2010, clone ST131 were more likely to have secondary bacteremia (OR, 5.05; 95% CI, 1.08 to 23.56) and non-use of the urinary catheter (OR, 3.77; 95% CI, 1.17 to 12.18; Chung et al., 2012). Additionally, another study involving 843 adults presenting with community-onset monomicrobial *E. coli* bacteremia at a medical center between 2008 and 2013 reported that more elderly (76.5 vs. 64.0%; *p* = 0.01) or nursing-home residents (12.7 vs. 3.8%; *p* < 0.001) were found in 102 adults infected by the ST131 clone in comparison to 741 adults by non-ST131 isolates (Wang et al., 2018). Although Chung et al. reported that the ST131 clone was not associated with higher mortality compared with the non-ST131 clone (Chung et al., 2012), Wang et al. found that the ST131 clone was associated with higher 28-day mortality, particularly in those infected by ESBL producers (Wang et al., 2018).

Among patients with UTI, ST131 *E. coli* still plays an important role in this clinical entity. A national surveillance analyzed the temporal trend of the ST131 clone among urinary *E. coli* isolates in the community from 2002 to 2016, in which 2,997 outpatient urine *E. coli* isolates were included and 542 were selected for detection of ST131 based on ciprofloxacin and/or cefotaxime resistance (Wang et al., 2021). Overall, the estimated ST131 prevalence gradually increased from 11.2% (in 2002–2004), 12.2% (in 2006–2008), 13.6% (in 2010–2012), to 17.4% in 2014–2016 (*p* < 0.01). In the ciprofloxacin-resistant/cefotaxime-resistant group, ST131 increased from 33.3% in 2002–2004 to 72.1% in 2014–2016 (*p* < 0.01). Moreover, age (≥65 years) and ciprofloxacin resistance were independently associated with ST131 (Wang et al., 2021).

#### 6.1.6. Pediatric patients

For children aged 0–18 years, a prospective study included 157 isolates from stool specimens collected within 3 days of hospitalization between 2013 and 2014 and showed that among 157 *E. coli* isolates, 26 (16.6%) and 13 (8.3%) were O25b and ST131 positive, respectively (Huang et al., 2018). Among 13 ESBL-producing *E. coli* isolates, five (38.5%) belonged to CTX-M group 9, among which 4 (80%) were CTXM-14 and O25b-ST131 positive (Huang et al., 2018). For infants <1 year, a study included infant patients hospitalized for ESBL-producing *E. coli*-associated UTI between 2009 and 2012 and found that O25b-ST131 accounted for 65% of the 111 isolates (Cheng et al., 2015). Although *E. coli* O25b-ST131 isolates were more susceptible to trimethoprim/sulfamethoxazole, there were more resistant to ciprofloxacin (Cheng et al., 2015).

#### 6.1.7. Environmental source

Several anthropogenic factors, including the drinking water process, pork consumption, pets, and household density might be associated with ST131 *E. coli* (Cheng et al., 2022). Compared with families who live in less crowded houses, participants with pets had a similar trend of higher risks of ESBL-producing *E. coli*, ST131 *E. coli*, and extraintestinal pathogenic *E. coli* fecal carriage among those living in houses accommodating relatively more people (Cheng et al., 2022). In addition to the above clinical settings, Chen et al. investigated the epidemiology of ESBL-producing *E. coli* from multiple rivers in southern Taiwan (Chen et al., 2016). They found that ESBL-producing *E. coli* mostly belonged to clonal complexes ST10 and ST58, which were geographically related to chicken farms, however, ESBL-producing *E. coli* ST131 was not detected among the isolates from river water (Chen et al., 2016).

#### 6.1.8. Animal sources

The ST131/O25b strain is a global zoonotic clone of public health concern, however, the prevalence of ST131 *E. coli* in animal studies was various. One study including 275 *E. coli* isolated from piglets with diarrhea in swine farms showed that the occurrence rate of ESBL-producing *E. coli* was 19.7% (*n* = 54) in southern Taiwan in 2015 (Lee and Yeh, 2017). The ST10 clonal complexes comprised most of the ESBL-producing *E. coli* strains and the most detected β-lactamase genes were the CTX-M-15 gene (16 of 54; 29.6%) and CTX-M-55 gene (34 of 54; 63.0%), which belong to the CTX-M-1 group (Lee and Yeh, 2017). Another surveillance screened for 283 *E. coli* isolates in dogs and cats from 2014 to 2017 and found 65 (23%) *E. coli* (54 from dogs and 11 from cats) with the ESBL phenotype (Huang Y. H. et al., 2020). Additionally, the CTX-M-1 group and CTX-M-2 group were the most identified ESBL gene groups. CTX-M-55 gene was the main ESBL gene within the CTX-M-1 group, whereas the CTX-M-2 group contained only CTX-M-124. Further multilocus sequence typing indicated that ST457, ST131, and ST648 were the most common sequence types, and eight ST131/O25b isolates were identified (Huang Y. H. et al., 2020). Another study investigated the rectal swab specimens from 299 non-infectious dogs and found that the prevalence of ESBL-producing *E. coli* was 9.4%, and seven isolates were positive for ST131, in which the most predominant subtypes were FimH41 and FimH22 (Chen et al., 2020). Overall, it is worth noting that the potentially wide-spreading CTX-M-55 has been emerging in *E. coli* isolates from both humans and animals in Taiwan (Jean and Hsueh, 2017).

### 6.2. Klebsiella pneumoniae

In Taiwan, *K. pneumoniae* was the prevalent pathogen in many types of infections, such as liver abscess, community-acquired pneumonia, lung abscess, empyema thoracic, urinary tract infections, spontaneous peritonitis, and peritoneal dialysis-related peritonitis (Chu et al., 1992; Wang et al., 2005; Lee et al., 2006; Chan et al., 2007; Lin et al., 2010b, 2014, 2015). However, the emergence of antibiotic-resistant *K. pneumoniae*, including ESBL-producing *K. pneumoniae* has largely limited the therapeutic options and posed a great threat to public health. Table 4 summarized the prevalence of ESBL-producing *K. pneumoniae* over time in Taiwan.

**Table 4:** Prevalence of extended-spectrum β-lactamases (ESBLs) among *Klebsiella pneumoniae* in Taiwan.

Study	Site and setting	Study period	No of isolates	Prevalence of ESBL-producing *K. pneumoniae*
Jan et al. (1998)	1 medical center in northern Taiwan	1993–1997	93	3.4% in 1993 to 10.3% in 1997
Liu et al., 1998	1 district teaching hospital in central Taiwan	1997	104	29.8%
Biedenbach et al. (1999)	6 medical centers in Taiwan	1999	51	21.7%
Lin et al. (2006)	Patient with *K. pneumoniae* liver abscess	1997–2001	30	23.3%
Hsueh et al. (2001)	ICUs in five centers in northern and southern Taiwan	2000	124	11.3%
Lin et al. (2003)	1 district hospital in northern Taiwan	2001	422	14%
Lee C. M. et al. (2009)	Respiratory care center in a medical center in northern Taiwan	2001–2002	53	69.1%
Kuo et al. (2007)	Children in a medical center in southern Taiwan	2000–2005	274	28.5%
Jean et al. (2009)	ICUs in 10 medical centers in northern, central and southern Taiwan	2005	162	26%
Tsai et al. (2010)	Diabetic patients with *K. pneumonia* bacteremia	2005–2006	193	45.7% (nosocomial group) 4.1% (community group)
Lee C. Y. et al. (2009)	Pediatric ICU in a medical center in central Taiwan	2001–2006	NA	20%
Lin et al. (2016)	25–28 regional hospitals and medical centers located in different geographical regions of Taiwan	2002–2012	1,016	4.8% (2002) to 11.9% (2012)

The prevalence of ESBL-producing *K. pneumoniae* has been higher in hospitals than in the community (Table 4). Although less reported for ESBL production in *E. coli*, the prevalence of ESBL-producing *E. coli* strains has been high in ICUs, respiratory care centers, and nursing homes in Taiwan (Table 3).

#### 6.2.1. Studies of prevalence before 2000

Three studies before 2000 had reported the prevalence of ESBL-producing *K. pneumoniae* isolates. In 1998, Jan et al. reported that the frequency of ESBL-producing *K. pneumoniae* isolates (according to the disk-diffusion method) had increased markedly in the years from 3.4% in 1993 to 10.3% in 1997 in a single center in northern Taiwan (Jan et al., 1998). At the same time, Liu et al. showed that 31 (29.8%) of 104 clinical isolates of *K. pneumoniae* collected over a period of 8 months were found to be ESBL producers in a district teaching hospital in central Taiwan in 1997 (Liu et al., 1998). In 1999, a multicenter study showed that ESBL production in *Klebsiella* spp. was found to be 21.7% from isolates in six medical centers in Taiwan (Biedenbach et al., 1999).

#### 6.2.2. Studies of prevalence between 2000 and 2010

Between 2000 and 2010, many studies in different parts of Taiwan or settings showed the various prevalence of ESBL-producing *K. pneumoniae*. A study in a district hospital in northern Taiwan found that 59 (14%) of 422 isolates of *K. pneumoniae* collected in 2001 were ESBL-producing strains (Lin et al., 2003). In addition, they demonstrated that tracheostomy and ceftazidime use were risk factors in the acquisition of *K. pneumoniae* with ESBLs (Lin et al., 2003). Moreover, a significantly rising prevalence of ESBL production among *K. pneumoniae* in national surveillance for 3 months in 2005 was noted when compared with a previous Taiwanese survey in 2000 (*p* = 0.002; Jean et al., 2009). Additionally, clonal dissemination (both interhospital and intrahospital dissemination) of ESBL-producing isolates of *K. pneumoniae* occurred in several regions of Taiwan, mainly in the hospitals located in the northern and central regions of Taiwan (Yu et al., 2004b).

For critically ill patients, a multicenter study in 2000 showed that the percentage of ESBL-producing *K. pneumoniae* was 11.3% of isolates from various clinical specimens from patients in ICUs (Hsueh et al., 2001). For pediatrics requiring ICU admission, a single medical center in central Taiwan showed that ESBL-producing *K. pneumoniae* accounted for 20% of *K. pneumoniae* isolates since 2005 (Lee C. Y. et al., 2009). In a respiratory care center, a retrospective analysis from January 2001 to December 2002 revealed the prevalence of ESBL phenotype was as high as 69.1% of 53 *K. pneumoniae* isolates (Lee C. M. et al., 2009). For patients with community-acquired liver abscess, a small study showed that seven of 30 *K. pneumoniae* strains associated with primary liver abscess were found to be ESBL-producers, in which SHV-5a was found in 5, whereas SHV-5 and CTX-M-9 group were detected in 1 strain (Lin et al., 2006). For diabetic patients, a retrospective analysis involving 193 adult diabetic patients with *K. pneumoniae* bacteremia hospitalized between January 2005 and December 2006 showed that the rate of ESBL infections in the nosocomial group was 11 times higher than that in the community group (45.7 vs. 4.1%, *p* < 0.001; Tsai et al., 2010). Moreover, ESBL infection accounted for 53% of mortality in the nosocomial group (Tsai et al., 2010).

#### 6.2.3. Studies of prevalence after 2010

One study including 57 fistula or graft- or catheter-related ESBL-producing *K. pneumoniae* bacteremia in patients on maintenance hemodialysis (HD) found most of the patients were elderly, malnourished, and with a history of severe illnesses, broad-spectrum antibiotic use before the onset of bacteremia, and severe septicemia (Yang et al., 2012). Further multivariate analyses revealed that flomoxef use (OR, 3.52; 95% CI, 1.19–58.17), Pitt bacteremia score (OR, 2.92; 95% CI, 1.36–6.26), and catheter-dependent HD >30 days (OR, 5.73; 95% CI, 1.21–63.2) were independently associated with increased mortality (Yang et al., 2012). A longitudinal study involving 1,016 *K. pneumoniae* isolates from the community- outpatients or those visiting emergency rooms collected during 2002–2012 from the Taiwan Surveillance of Antimicrobial Resistance program showed that the prevalence of ESBL-producers significantly increased from 4.8% in 2002 to 11.9% in 2012 (*p* = 0.012; Lin et al., 2016).

#### 6.2.4. Genotypes of ESBL in *Klebsiella pneumoniae*

In 1998, Liu et al. first reported the molecular epidemiology of ESBL-producing *K. pneumoniae* clinical isolates in Taiwan and found that 22 (71%) of 31 isolates were found to produce SHV-5 (Liu et al., 1998). Additional analytical isoelectric focusing also found seven isolates that produced two unknown ESBLs (with pIs of 7.9 and 7.75), and the pI of 7.9 was later presumed to be CTXM-14 (Liu et al., 1998). In 2000, Yan et al. detected 20 (8.5%) ESBL producers among 234 nonrepetitive clinical isolates of *K. pneumoniae*, in which a predominance of SHV-12 in 10 strains followed by SHV-5 in 4 strains (Yan et al., 2000). In 2001, Chang et al. repdqorted the stepwise mutations initiated from SHV-1 or SHV-11 into SHV-2, SHV-5, and SHV-12 comprise the evolutionary change responsible for ESBL production based on the analysis of a total of 113 blood culture isolates of *K. pneumoniae* from 10 hospitals in northern Taiwan (Chang et al., 2001).

#### 6.2.5. Transmission mechanisms of the ESBL enzymes

In 2002, a multicenter study first identified another rapidly evolving group of CTX-M family enzymes (CTX-M-3 and CTX-M-14) with inter-and intrahospital clonal dissemination in Taiwan (Yu et al., 2002). In 2004, CTX-M-15, an Asp-240-Gly variant of CTX-M-3 with increased catalytic efficiency against ceftazidime, was first described in two of 211 ESBL-producing *K. pneumoniae* isolates (Yu et al., 2004a). In 2006, Yu et al. found that the insertion sequence IS26 and IS5 were found downstream from the SHV-5 gene in a transferable plasmid pKP53 (Yu et al., 2006a). In 2009, nationwide surveillance from seven medical centers and 13 regional hospitals showed that 102 (43.4%) of 235 ESBL-producing *K. pneumoniae* isolates were resistant to amikacin (Ma et al., 2009). Moreover, 92 of these 102 (90.2%) isolates were carrying CTX-M-type β-lactamases individually or concomitantly with SHV-type or CMY-2 β-lactamases and CTX-M-type β-lactamase genes belonging to either group 1 (CTX-M-3 and CTX-M-15) or group 9 (CTX-M-14) were found in all amikacin-resistant ESBL-producing *K. pneumoniae* isolates (Ma et al., 2009). Further molecular typing revealed that the amikacin-resistant ESBL-producing *K. pneumoniae* isolates were epidemiologically unrelated, and suggested that the acquisition of resistance was not through the spread of a resistant clone (Ma et al., 2009). In 2017, Chang et al. included 51 *E. coli* transconjugant strains with plasmids from ESBL-producing *K. pneumoniae* from the Taiwan Surveillance of Antimicrobial Resistance III Program and found that all the 51 plasmids carried a CTX-M gene, the majority of which were CTX-M-3 gene [28/51 (54.9%); Chang et al., 2017]. They found that the most common replicon type of plasmids was incompatibility group (Inc)A/C (60.8%), in which all carried CTX-M-3, CTX-M-14, and CTX-M-15 genes, and some also carried SHV-5 and SHV-12 genes (Chang et al., 2017). In this surveillance, greater than 50% of plasmids fell into clusters, and >60% of cluster-classified plasmids were present in clonally unrelated isolates, which should suggest that horizontal transfer of plasmids in the spread of ESBL genes (Chang et al., 2017).

### 6.3. Enterobacter cloacae complex

*Enterobacter cloacae* complex is an important nosocomial pathogen. One study in central Taiwan demonstrated that ESBL-producing strains could be associated with higher mortality than non-ESBL strains in patients with bloodstream infections (Chen and Huang, 2013). Furthermore, they found that the risk factors for ESBL among *E. cloacae* complex isolates included diseases severity (*p* = 0.03), category of healthcare-associated infection (*p* = 0.04), prior use of antibiotics (*p* = 0.023), and prior use of a ventilator (*p* = 0.037; Chen and Huang, 2013). One study tested a total of 116 clinical isolates of *E. cloacae* complex in northern Taiwan and found that the overall prevalence of ESBL-producing *E. cloacae* complex was 21.6% (Yang et al., 2009). Similar findings were shown in another study in a medical center in southern Taiwan, in which ESBL producers were identified in 20 (27.0%) of 74 clinical isolates by polymerase chain reaction-based methods (Su et al., 2010). Additionally, one more study in southern Taiwan showed that 17 of 110 *E. cloacae* complex isolates were ESBL producers, and further gene analysis reveal the presence of the SHV-12 gene in all ESBL producers (Yu et al., 2006b). In addition, one and two isolates carried the CTX-M-3 gene and CTX-M-9 gene, respectively. However, no major epidemic clone of ESBL producers was identified by pulsed-field gel electrophoresis (Yu et al., 2006b). In ICU, a study conducted at four ICUs of a tertiary hospital showed that SHV-12 (59%), CTX-M- 3 (36%), and CTX-M-14 (14%) were the three most frequent ESBLs among a total of 125 nonrepetitive ESBL-producing isolates of *E. cloacae* complex, *E. coli*, and *K. pneumoniae* (Wu T. L. et al., 2006). Furthermore, SHV-12 was predominant among the *E. cloacae* complex in the burn unit (Wu T. L. et al., 2006). A large study of 610 *E. cloacae* complex bacteremic isolates in a medical center during an 8-year period showed that 138 (22.6%) carried ESBL genes (Lee et al., 2010). In addition, 133 (96.3%) carried the SHV-12 gene, 3 (2.1%) had CTX-M-3, and 2 (1.4%) had both the SHV-12 and CTX-M-3 genes (Lee et al., 2010). Mover, they observed that the in-hospital sepsis-related mortality rate of patients definitively treated with a carbapenem was lower than that of those treated by non-carbapenem β-lactams (5/53, or 9.4%, vs. 13/44, or 29.5%; *p* = 0.01), and suggested that the survival benefit by carbapenem therapy for ESBL-producing *E. cloacae* complex bacteremia may provide therapeutic benefits (Lee et al., 2010).

### 6.4. Serratia marcescens

The study investigating the ESBL-producing *Serratia marcescens* is limited. One single-center study reported that ESBL producers could be identified in 11 (15.9%) of 69 *S. marcescens* isolates collected from patients hospitalized at a medical center in southern Taiwan from 1999 to 2002 by polymerase chain reaction-based methods (Su et al., 2010). In ICU, a national surveillance reported that ESBL production was found in 16% of *S. marcescens* isolates (Jean et al., 2009). The clinical spectrum of ESBL-producing *S. marcescens-*related infections included urinary tract infection; pneumonia, spontaneous bacterial peritonitis, secondary bacteremia, primary bacteremia, and colonization of the central catheter tip (Cheng et al., 2006). The 30-day mortality rate of ESBL-producing *S. marcescens*-related infections was 33.3% (5/15) in one study, in which their outcome varied according to the severity of the underlying disorder and the site of infection (Cheng et al., 2006). Among 123 nonrepetitive *S. marcescens* clinical isolates, 15 (12%) were ESBL-producers with exclusively revealing CTX-M-3 (Cheng et al., 2006). A similar finding was shown in the survey in a medical center in middle Taiwan, which found that CTX-M-3 was the most common ESBL gene in *S. marcescens* isolates (Wu et al., 2004).

### 6.5. Proteus mirabilis

ESBL-producing *P. mirabilis* is another common *Enterobacterales* in Taiwan. In central Taiwan, a two-center study reported that 34 (30.6%) of 111 clinical isolates of *P. mirabilis* from patients with respiratory or urinary tract infection had ESBLs, in which CTX-M-14 in 33 strains and CTX-M-3 in 6 strains (5 strains harboring both CTX-M-14 and CTX-M-3 enzymes; Wu L. T. et al., 2006). This study was also the first one to demonstrate the clonal spreading (both intra- and interhospital spread) of CTX-M-type *P. mirabilis* (Wu L. T. et al., 2006). These findings were consistent with another study in central Taiwan including 314 *P. mirabilis* isolates from urine, in which 79 (25%) clinical isolates were ESBL-producing and most ESBL-producing *P. mirabilis* isolates were positive for CTX-M (Chen C. M. et al., 2017). Additionally, they found that class 1 integrons were more frequently found in ESBL-positive (55/79, 70%) than ESBL-negative (21/235, 8.9%) *P. mirabilis* isolates (Chen C. M. et al., 2017). However, the study in southern Taiwan found a lower prevalence of ESBL among *P. mirabilis*, which was 2.8% (44/1574) but it increased from 0.7% in 1999 to approximately 6% after 2002 (Wu et al., 2008). Nonetheless, they showed a similar finding that all ESBL-producing *P. mirabilis* were positive for CTX-M, including 22 CTX-M-14, 18 CTX-M-3, two CTX-M-24, and two CTX-M-66 producers (Wu et al., 2008). In an additional study focusing on nursing home residents in southern Taiwan, the prevalence of ESBL in 102 *P. mirabilis* isolated was 4.9% (*n* = 5; Liu et al., 2016). Two nationwide surveillance showed similar findings that the prevalence of ESBL was 8.2% in 25 to 28 hospitals between 2002 and 2012 (Wang et al., 2014), and 13% in *P. mirabilis* isolates at the ICUs of 10 major teaching hospitals in 2015 (Jean et al., 2009). As previously described (Wu L. T. et al., 2006; Wu C. M. et al., 2008; Chen et al., 2017), the CTX-M type remained the predominant ESBL gene in the nationwide study (Wang et al., 2014).

## Conclusion

The prevalence of ESBL in *Enterobacterales* has been increasing over time in Taiwan, however, the prevalence of ESBL could be various. The rates of ESBL producers have been highest in northern Taiwan. The genotypes of ESBL are also different in various studies in Taiwan. SHV-type ESBLs (SHV-5 and SHV-12) were the major type of *E. cloacae complex*, but *S. marcescens*, *E. coli*, and *K. pneumoniae* were more likely to possess CTX-M-type ESBLs (CTX-M-3 and CTX-M-14). A clonal sequence type of O25b-ST131 among urinary or bloodstream *E. coli* isolates, potentially associated with virulence, ESBL production, ciprofloxacin resistance, and mortality has been emerging in the community in Taiwan. The evolution of the genetic traits of the ESBL-producing *Enterobacterales* isolates has established clonal dissemination (both interhospital and intrahospital dissemination) in several regions of Taiwan. The cause of different prevalence and genotypes of ESBL in *Enterobacterales* in different regions might be due to different hospital allocation, clinical specimens, sources of infections, antibiotic prescription by physicians, and nosocomial or community acquisitions. Continuous monitoring and surveillance in the investigation of ESBL production among *Enterobacterales* are needed to establish its long-term epidemiology.

## Author contributions

C-MC, C-CL, and W-LY contributed to conception and design of the study. C-MC and C-CL wrote the first draft of the manuscript. W-LY critically reviewed the manuscript. All authors contributed to the article and approved the submitted version.

## Conflict of interest

The authors declare that the research was conducted in the absence of any commercial or financial relationships that could be construed as a potential conflict of interest.

## Publisher’s note

All claims expressed in this article are solely those of the authors and do not necessarily represent those of their affiliated organizations, or those of the publisher, the editors and the reviewers. Any product that may be evaluated in this article, or claim that may be made by its manufacturer, is not guaranteed or endorsed by the publisher.
